# A cantilever-beam-structured triboelectric nanogenerator for micro-vibration energy harvesting

**DOI:** 10.1016/j.isci.2026.115690

**Published:** 2026-04-13

**Authors:** Wenxuan Chang, Hengyu Guo

**Affiliations:** 1 State Key Laboratory of Mechanical Transmission, College of Mechanical and Vehicle Engineering, Chongqing University, Chongqing 400044, China; 2 School of Physics, Chongqing University, Chongqing 400044, China

**Keywords:** Sensor, Energy application, Devices

## Abstract

Triboelectric nanogenerator is a new type of self-powered device that can harvest vibrational energy from the environment. Harvesting low-frequency micro-vibration energy remains challenging for conventional approaches. This study presents a cantilever-beam triboelectric nanogenerator (CB-TENG) that combines resonant mechanical amplification with integrated voltage-multiplying circuitry to enhance energy conversion. The device utilizes a resonant cantilever beam to mechanically amplify micro-scale displacements into effective contact-separation motions, integrated with a voltage-multiplying circuit for enhanced charge accumulation. The results show that the CB-TENG significantly outperforms a comparable piezoelectric bimorph in output power under micro-vibrations (30–90 μm). The TENG can be used to collect low-frequency micro-vibration energy to power equipment operating condition monitoring and sensing networks. It successfully powers commercial electronic devices, such as LEDs and a hygrothermograph, showcasing its potential as a practical power source for equipment condition monitoring and self-powered sensing networks.

## Highlights

• A cantilever beam structure is used to harvest micro-vibration energy
• CB-TENG achieves higher energy density compared to traditional PENG
• Introduce a power management system to increase power density

## Graphical abstract

**Figure:**
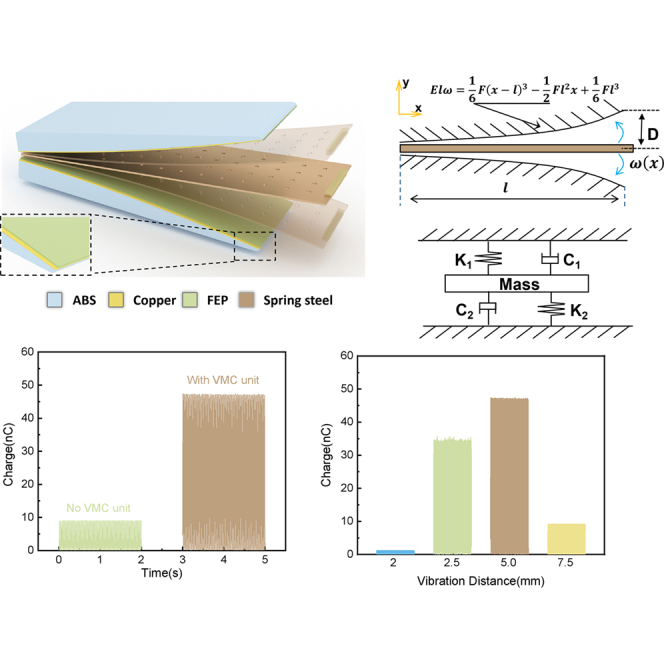

## Introduction

The rapid development of the internet of things (IoT) has led to ubiquitous sensors that bring great convenience to daily life.^1^^,^^2^^,^^3^ The IoT has been integrated into our lives from every aspect, and the concept of internet of everything is constantly emphasized, bringing radical changes and development in our society.^4^ However, power supply remains a critical bottleneck limiting the large-scale deployment of sensor nodes.^5^ Currently, most of the IoT end nodes are battery-powered, but the batteries need to be regularly maintained or replaced, which is extremely costly, and the disposal of old batteries will bring great pressure to the ecological environment.^6^^,^^7^ Wired power is not a viable large-scale solution due to constraints related to deployment environment, material and labor costs, and ongoing maintenance. Thus, future IoT terminals and sensors require more stable, continuous, and reliable power solution.

Triboelectric nanogenerator (TENG), a novel mechanical energy harvester based on the coupling of contact electrification and electrostatic induction invented by Wang’s group in 2012, has been proved to be well adapted to low frequency energy.^8^^,^^9^^,^^10^^,^^11^ TENG has shown great potential for self-driven sensing due to its low cost,^12^ simple manufacturing process,^13^ and good scalability.^14^ TENG can be used to efficiently harvest wind,^15^ ocean,^16^ and vibration energy^17^ to power various electronic devices has attracted a vast attention.^18^^,^^19^ While mechanical vibration in the environment is ubiquitous, extracting vibration energy from the environment to power sensor devices has a broad application prospect. In terms of low-frequency mechanical energy, triboelectric nanogenerators (TENG) can effectively convert vibration energy into electrical energy to power IoT sensors.^20^^,^^21^^,^^22^ Micro-vibration sensors have the advantages of small size,^23^^,^^24^ light weight,^25^^,^^26^ fast response,^27^ high sensitivity,^28^ and low cost,^29^ which have far-reaching effects on signal detection systems in different applications, such as aviation,^30^ remote sensing,^31^ medical,^32^ and industrial automation.^33^^,^^34^ Notably, a cantilever beam structure triboelectric nanogenerator is proposed to achieve vibration energy harvesting in the presence of micro-vibrations. The traditional piezoelectric cantilever beam structure converts vibration energy into electrical energy.^35^ The piezoelectric conversion method is simple and has relatively high voltage and power density, long life cycle, and very wide application prospects.^36^^,^^37^ However, the piezoelectric effect requires substantial material deformation. Under micro-vibrations, changes in electric dipole polarization are minimal, limiting output.^38^ In contrast, TENGs can achieve efficient energy harvesting at micro-displacements through ingenious structural design, as evidenced by numerous prototypes.

In this study, we designed a triboelectric nanogenerator based on a cantilever beam structure, which was compared with a piezoelectric cantilever beam structure in the micro-vibration case to achieve a higher output power of the friction nanogenerator under tiny vibrations. By amplifying micro-vibrations through a cantilever beam structure and converting them into periodic contact-separation motion, micro-vibration energy is effectively harvested. Perforated spring steel plates function as electrodes, with the fluorinated ethylene propylene (FEP) on the support acting as the triboelectric layer, collecting micro-vibration energy through cantilever beam oscillation. A dedicated voltage-multiplying circuit (VMC) is integrated to achieve continuous charge accumulation, overcoming the inherent charge saturation limit and significantly boosting the effective output power. With the introduction of a power management system and using the cantilever beam electrode as the excitation electrode, the TENG achieves a rapid increase in charge density. This work demonstrates that our CB-TENG, operating at resonance (33 Hz), achieves an output power of 1.755 mW under 50 μm excitation, which is 2.03 times higher than a comparable piezoelectric bimorph. It successfully powers 70 LEDs and a hygrothermograph, validating its potential as a practical power source for equipment condition monitoring and wireless sensing networks. TENG can achieve efficient energy harvesting in the case of small displacements.

## Results

### Structure and working principle of CB-TENG

**Figure 1:**
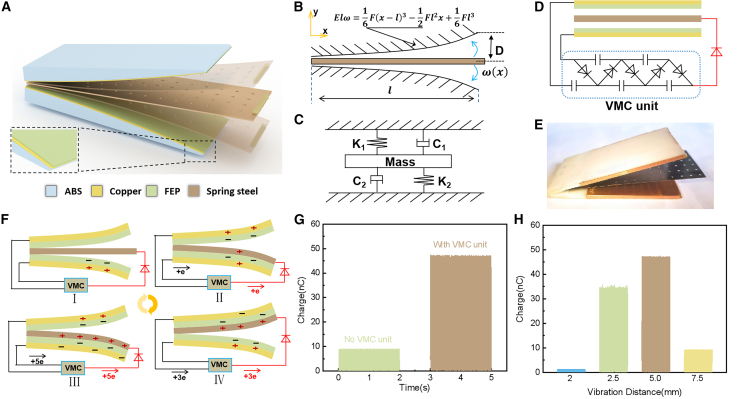
Structure schematic and working mechanism of the CB-TENG (A) 3D structural diagram of the CB-TENG. (B) Model of the proposed cantilever beam nonlinear generator. (C) The equivalent vibration schematic of the model can be considered as two spring damping system. (D) Voltage-multiplying circuit (VMC) detailed circuit diagram. (E) Digital photograph of CB-TENG. (F) The working mechanism of CB-TENG. (G) Variation of CB-TENG charge under different circuit connections. (H) The charge of CB-TENG at 33 Hz, 50 μm vibration conditions (2.0, 2.5, 5, and 7.5 mm).

Figure 1A shows the 3D schematic diagrams of the CB-TENG device, which consists of two supports and spring steel sheets. 3D printer prints the arc shaped module as the support part, with a thickness of 0.01 mm copper tape as the electrode on the surface, and covered with a thickness of 30 μm FEP film as the electrical layer. The spring steel sheets of the cantilever beam structure are sandwiched in the middle, which is forced to vibrate and contact with the upper and lower electrification layers respectively, and make up two sets of contact separation mode cantilever beam structure triboelectric nanogenerator to collect vibration energy. The cantilever beam structure TENG works by providing micro vibration from the actuating vibration table to convert the low frequency micro vibration energy into higher frequency vibration energy to achieve micro displacement vibration energy collection. The output is increased by power management to realize charge accumulation. Figure 1B shows the model of the proposed cantilever beam nonlinear generator, D is the vibration distance of the cantilever beam structure TENG, and L is the length of the cantilever beam.

(Equation 1) $$F=\frac{WEDT^{3}}{4L^{3}}\text{,}$$

Where F is the theoretical forward force and W is the width of the cantilever beam, T is the thickness of the cantilever beam, E is the modulus of elasticity of the steel sheet, $E=2100000\;N/{mm}^{2}$.

The approximate differential equation to calculate the cantilever beam deflection ω(x) by the cantilever beam equation is that,

(Equation 2) $$\frac{d^{2}\omega }{dx^{2}}=\frac{M\left(x\right)}{EI}\text{,}$$

The deflection equation is obtained after integrating twice,

(Equation 3) $$EI\omega =\iint M\left(x\right)dxdx+C_{x}+D=\frac{1}{6}F{\left(x-l\right)}^{3}+C_{x}+D\text{.}$$

Apply the boundary conditions, and the following formula can be yielded,

(Equation 4) $$C=-\frac{1}{2}Fl^{2},D=\frac{1}{6}Fl^{3}$$

(Equation 5) $$EI\omega =\frac{1}{6}F{\left(x-l\right)}^{3}-\frac{1}{2}Fl^{2}x+\frac{1}{6}Fl^{3}$$

(Equation 6) $$\omega_{\max}=\frac{Fl^{3}}{6EI}\text{.}$$

Where l is the effective length of the cantilever beam, I is the cross-sectional area of the steel sheet, $I=30\times 0.15=4.5\;{mm}^{2}$. c and d are the integration constants. When the actuating vibration table applies a positive force F to the CB-TENG, the cantilever beam is deflected to contact with the fixture model to realize the contact separation triboelectric nanogenerator.

Figure 1C is a conceptual diagram of the structure, where the contact separation of TENG is achieved by collecting micro vibration energy to induce cantilever beam resonance, analogous to the vibration state of the spring. The equivalent vibration schematic of the model can be considered as two spring damping system. Figure 1D realize excitation through a voltage-multiplying circuit (VMC). VMC consists of five diodes and ceramic capacitors. Figure 1E shows the photo dimensions of each part of the device. The spring steel sheet of cantilever beam structure is 80 mm in length, 30 mm in width, and 0.15 mm in thickness. The electrostatic adherence caused by air resistance is counteracted by perforating holes in the spring steel sheet. The difference of output is shown in supplemental information Figure S1. When the TENG is operating, the rapid vibration causes the air resistance on both sides of the spring steel sheet to increase, preventing it from moving to the other end, and the surface charge of the steel sheet gradually increases, which is attracted to attach to the bracket model due to the electrostatic induction phenomenon. Figure 1F shows the working principle of the CB-TENG. A VMC system is introduced to achieve charge accumulation and improve output performance. The VMC diagram is shown in Figure S2, which consists of five diodes and five capacitors. Taking advantage of the characteristics of the diode unidirectional conduction (equivalent to a switch) and the properties that the voltage across the capacitor unable to change abruptly and can store energy, the energy is gradually transported to the later stage, and the voltage in the system is gradually increased. The VMC is used to achieve charge accumulation and increase output. The CB-TENG cycle operation charge accumulation process and the working principle of VMC unit is shown in Method S1. In the initial state, the system is in equilibrium. When the CB-TENG is excited for periodic contact separation motion, the VMC unit switches series and parallel connection, generating alternate charging and discharging to achieve charge accumulation, and the charge of Cu electrode gradually increases. The VMC unit starts charging, and the output voltage increases with the increase of operation cycle, and the charge of spring steel sheet electrode reaches the saturation state, and the output voltage reaches stability. Figures 1G and S3 indicates the output under different operating conditions, the output of VMC unit is 5 times more than without VMC. It shows that efficient output can be achieved through the VMC unit. Figure 1H shows the output of CB-TENG at 33 Hz, 50 μm vibration conditions, where the vibration distance is 2.0 mm, 2.5 mm, 5 mm, and 7.5 mm, and the cantilever beam length is 40 mm. When the vibration distance is 2.0 mm and 7.5 mm, the output is shown in Figure S4. Under the vibration distance is 2.0 mm, the spring steel sheet is forced to vibrate and continues to move even after contacting the dielectric layer. The vibration of the spring steel sheet is damp and the vibration energy decays in the reciprocal motion, while the spring steel sheet is subjected to electrostatic adsorption and adsorbed to the bracket model. When the vibration distance is 7.5 mm, the cantilever beam system is operating at 50 μm vibration conditions, which is receiving a forward force unable to drive the cantilever beam structure into complete contact with the support model, the output is reduced.

(Equation 7) y(x,t)=Y(x)cosωt,

where y(x,t) is the displacement curve function of the spring steel sheet. Y(x) is the displacement of the spring steel piece at x, ω is the frequency of vibration, and t represents the time.

The equation of motion of the cantilever beam has the expression as,

(Equation 8) $$EI\frac{\partial^{4}y}{\partial x^{4}}+m\frac{\partial^{2}y}{\partial t^{2}}=0\text{,}$$

Boundary conditions of cantilever beam:

$$y\left(x=0\right)=0,\frac{dy}{dx}\left(x=0\right)=0,{\;\frac{\partial^{2}y}{\partial x^{2}}|}_{x=l}=7,{\frac{\partial }{\partial x}\left(EI\frac{\partial^{2}y}{\partial x^{2}}\right)|}_{x=l}=m\text{;}$$

(Equation 9) y(x,t)=Y(x)T(t),

Substituting into the equation of motion of the cantilever beam is obtained as,

$$Y\left(t\right)=C_{1}\;\cos\;\beta x+C_{2}\;\sin\;\beta x+C_{3}\;\cosh\;\beta x+C_{4}\;\sinh\;\beta x\text{;}$$

(Equation 10) T(t)=Acosωt+Bsinωt.

Since the maximum displacement of the cantilever beam structure is 7 mm. During the vibration, the spring steel sheet cannot fully contact the support model.

The motion of the spring steel sheet is determined by the external load. The spring steel sheet resonance frequency is adjusted to 33 Hz. According to the spring steel sheet equation of motion and the Euler-Bernoulli beam equation combined to obtain the curve equation, the vibration curve of the cantilever beam is determined by its width, length, and thickness.

### Output performance of the piezoelectric bimorph and CB-TENG

**Figure 2:**
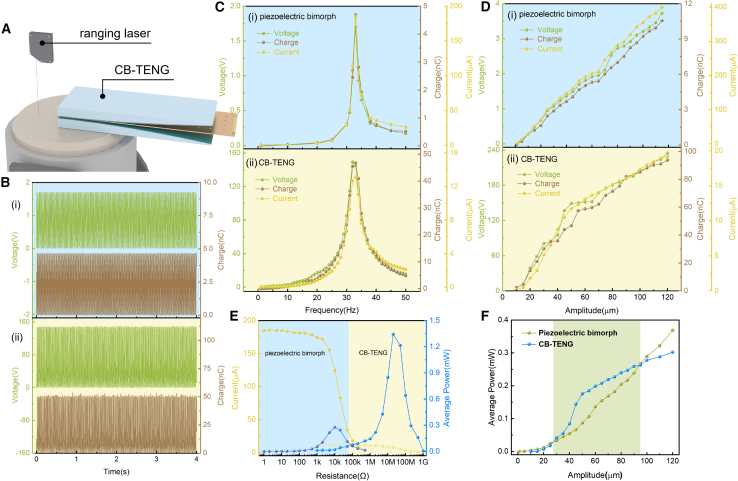
The performance of piezoelectric bimorph and CB-TENG (A) The 3D schematic diagrams of the actuating vibration table and TENG device. (B) The voltage and charge of (1) a piezoelectric bimorph and (2) CB-TENG under 33 Hz at 50 μm amplitude. (C) The output of (1) piezoelectric bimorph and (2) CB-TENG at different frequencies. (D) The output of (1) the piezoelectric bimorph and (2) the CB-TENG as a variation curve of vibration amplitude under 33 Hz. (E) The average power and current of the piezoelectric bimorph and CB-TENG under various impedances. (F) Comparison of the average power of the CB-TENG and the piezoelectric bimorph under micro displacements of 30–90 μm.

With the rapid development of wireless sensing energy, vibration energy harvesting has great potential and broad application prospects. Currently, piezoelectric conversion energy harvesting is widely used to convert vibration energy into electrical energy through piezoelectric effect. Traditional piezoelectric materials are made of brittle materials with a limited force tolerance range, easily fatigued and unrecoverable for long working hours, and have a short service life and need to be replaced frequently, which limits the development of their applications. Triboelectric nanogenerators can effectively convert vibration energy into electrical energy to power IoT sensors. Figure 2A shows the 3D schematic diagrams of the actuating vibration table and TENG device. The photograph of a piezoelectric bimorph is shown in Figure S5. The effective length of the piezoelectric bimorph is 60 mm and the width is 30 mm. When the actuating vibration table to generate excitation, the piezoelectric bimorph is forced to vibrate and subjected to inertial forces, the piezoelectric cantilever beam is deformed, due to the piezoelectric effect, generates a potential difference. The piezoelectric bimorph produces a stable output due to deformation. Figure 2B(1) shows the output voltage of a piezoelectric bimorph with a frequency of 33 Hz at a micro vibration of 50 μm is 1.71 V and the output charge is 4.66 nC. Figure 2B(2) and Video S1 shows the output at 33 Hz, 50 μm for a small vibration case, which reaches 52.37 nC and 149.124 V. Figure 2C and Video S2 shows the output of (1) piezoelectric bimorph and (2) CB-TENG at different frequencies. The performance of piezoelectric bimorph (Figure S6) and CB-TENG (Figure S7) at different frequencies (1) 10 Hz, (2) 30 Hz, (3) 33 Hz, (4) 35Hz, and (5) 40 Hz, respectively. The output gradually increases as the frequency gradually increases, and the piezoelectric bimorph reaches resonance at 33 Hz with maximum output. As the frequency continues to increase, the output gradually decreases. When the piezoelectric bimorph gradually approaches the resonant frequency, the forced vibration distance gradually increases, the deformation of the piezoelectric bimorph becomes larger, and due to the piezoelectric effect, the piezoelectric sheet is polarized and the electric field is generated, and the output gradually increases. As the frequency continues to increase, the vibration distance gradually decreases, the piezoelectric sheet deformation becomes smaller, and the output gradually decreases. The resonant frequencies of the cantilever beam structure TENG using the Dunkley method.

(Equation 11) $$\sum_{i=1}^{n}\frac{1}{p_{i}^{2}}=\frac{1}{p_{1}^{2}}+\frac{1}{p_{2}^{2}}+\cdots +\frac{1}{p_{n}^{2}}\text{,}$$

Where *p* is the fundamental frequency, When m_1_ exists alone,

(Equation 12) $$p_{1}^{2}=\frac{k}{m}\text{.}$$

Video S1. The output at 33 Hz for CB-TENG

Video S2. The output at 33 Hz for piezoelectric bimorph

Substitute Dunkley method formula, $p_{1}=0.3535\sqrt{k/m}$. The TENG resonance frequency was adjusted in 33Hz by adjusting the counterweight of the cantilever beam structure. The output of the CB-TENG increases and then decreases at different frequencies, with the maximum output at 33 Hz. At the resonant frequency of 33 Hz, the forced vibration distance is maximized and the effective contact area of the CB-TENG is maximized, producing the highest output. When the frequency is changed, the contact area decreases and the output reduces. Figure 2D shows the output of (1) the piezoelectric bimorph and (2) the CB-TENG as a variation curve of vibration amplitude under 33 Hz. The performance of piezoelectric bimorph (Figure S8) and CB-TENG (Figure S9) at different frequencies (1) 10 mm, (2) 30 mm, (3) 50 mm, (4) 70 mm, and (5) 90 mm, respectively. In the case of micro displacement vibration, the output of the piezoelectric bimorph and the CB-TENG increases as the amplitude gradually increases. As the amplitude gradually increases, the distance at which the piezoelectric bimorph is forced to vibrate also increases, and greater deformation occurs, and the output gradually increases. The distance of the CB-TENG forced to vibrate also increases, the effective contact area increases and the output gradually increases.

Figure 2E shows the matched impedance of a piezoelectric bimorph and CB-TENG with a load resistance. The maximum output power of piezoelectric bimorph is 0.387 mW at 33 Hz with a matched load of 10 kΩ and the power performance of CB-TENG under 33 Hz, 50 micron vibration conditions with different external load resistances reaches a maximum output power of 1.88 mW when the load resistance is 20 MΩ. By comparing the output power of cantilever beam structure TENG and piezoelectric bimorph under micro displacement is shown in Figure 2F, it is found that the cantilever beam structure TENG has higher output power under the micro displacement of 30–90 μm. The vibration amplitude of the actuating vibration table is adjusted to 50 μm by laser ranging is shown in Supporting Information Figure S10 and Video S3. In the case of 50 μm micro vibration displacement, the output power of CB-TENG is 0.1754 mW, which is 2.1 times higher than that of piezoelectric bimorph.

Video S3. The vibration amplitude of the actuating vibration table is adjusted to 50 μm by laser ranging

**Figure 3:**
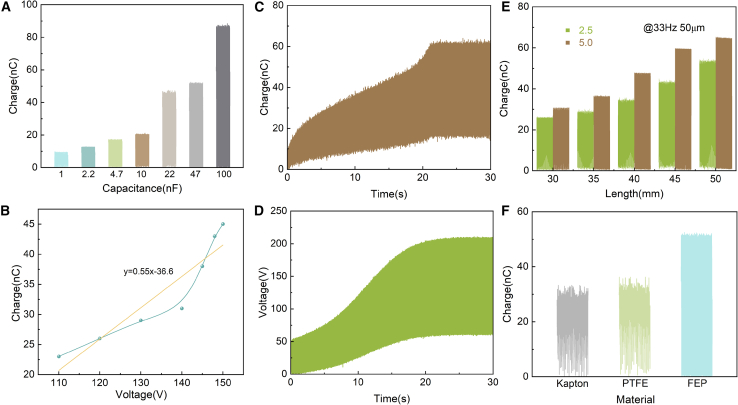
The performance of CB-TENG (A) The transferred charges with different capacitors. (B) The relationship between transfer charge and voltage. (C) The output of transfer charge. (D) The output of voltage. (E) The output of CB-TENG, where the vibration distance is 2.5 mm and 5 mm, and the cantilever beam length is 30–50 mm. (F) The charge output of different materials.

The influence of circuit parameters and structural design on the output of the CB-TENG was systematically investigated. As shown in Figure 3A, the saturation charge transferred by the excitation circuit increases with larger capacitance values, albeit at the cost of a longer accumulation time (See Figure S11 for complete curves). The relationship between transferred charge and voltage when using a 47 nF capacitor exhibits an initial linear rise followed by saturation (Figure 3B).

The effect of different capacitors in the excitation circuit on the CE-TENG output is systematically discussed below. Theoretically, the amount of charge transferred between the main-TENG and the capacitor gradually increases as the capacitor capacity increases. As shown in Figure 3A, the transferred charges when the excitation circuit reaches stability with different capacitors (1.0, 2.2, 10, 22, 47, and 100 nF, respectively). As the value of capacitance in the circuit increases, the output also increases. When a 47 nF capacitor is used for the excitation circuit, the relationship between transfer charge and voltage is shown in Figure 3B, where the transfer charge rises linearly with increasing voltage at the beginning and then saturates. The size of the capacitance in the excitation circuit determines the time it takes for the charge to reach saturation output; the larger the capacitance value, the longer the accumulation time and the more charge transferred. It is clear from the test that if the capacitance value is small, the transferred charge each vibration cycle also decreases. The effect of different capacitors in the excitation circuit on the CE-TENG output is systematically discussed further. The effect on the process of charge accumulation by adjusting the size of the capacitance of the VMC unit are presented in Figure 3A. The charge grows exponentially, achieving rapid charge accumulation to an equilibrium state with the VMC unit.

(Equation 13) $$Q_{n+1}=Q_{2}{\left(1+\frac{C}{2C_{0}+C}\right)}^{n-1}\;\left(\;n\geq 2\right)$$

Where Q is the charge of the TENG, n is the number of contact separation movements, C is the capacitance of the TENG, and $C_{0}$ is the capacitance of the capacitor in the circuit.

Under different capacitance values (1, 2.2, 4.7, 10, 22, 47, and 100 nF, respectively), complete charge accumulation curves are shown in Figure S11, respectively. The transferred charge gradually increases when the excitation circuit reaches stability with different capacitors. When using 1.0 nF capacitors, the saturated charge is 9.47 nC and the charge accumulation time is merely 1.46 s. At a 100 nF capacitor with a saturation charge of 87.66 nC, the charge accumulation time has increased to 39.728 s. The stabilization time gradually increased from 1.46 s to 39.728 s. The output charge gradually increases as the capacitance value increases. However, the charge accumulation time is significantly expanding, which means that the charge accumulation rate is significantly lower. When a 47 nF capacitor is used for the excitation circuit, the relationship between transfer charge and voltage is shown in Figures 3C and 3D, where the transfer charge rises linearly with increasing voltage at the beginning and then saturates. The size of the capacitance in the excitation circuit determines the time it takes for the charge to reach saturation output; the larger the capacitance value, the longer the accumulation time and the more charge transferred. It is clear from the test that if the capacitance value is small, the transferred charge each vibration cycle also decreases. Figures 3E and S12 shows the output of CB-TENG at 33 Hz, 50 μm vibration conditions, where the vibration distance is 2.5 mm and 5 mm, and the cantilever beam length is 30–50 mm. As the cantilever beam length increases, the output also increases. This is because the longer cantilever beam increases the effective contact area of the triboelectric nanogenerator, thus enhancing the output. Figures 3F and S13 displays the transfer charge with different materials. The output performance of CB-TENG with three types of materials is studied at 33 Hz, 50 μm vibration conditions, 40 mm cantilever beam length, and 2.5 mm vibration distance. Due to the difference in the electronegativity of the materials, the accumulation charges vary, the charge output increase and stabilize to 32.166 nC, 36.584 nC, and 52.37 nC, respectively. Moreover, different materials require varying times to reach saturation, Kapton takes 12.1 s, PTFE takes 18.564 s, but FEP achieves stable charge in just 22.12 s. It explains that the transfer charge is largest with FEP film due to a higher triboelectronegativity.

### Application demonstrations of CB-TENG

**Figure 4:**
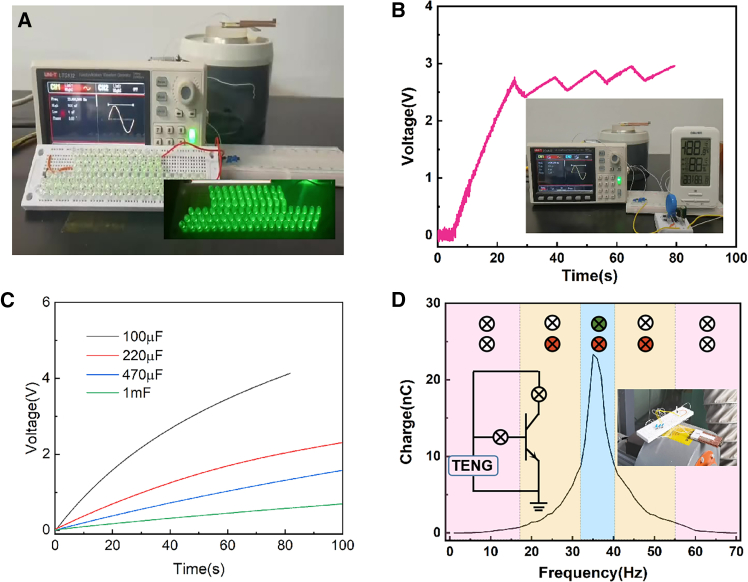
Application demonstrations of CB-TENG (A) Photograph of 70 LEDs lighted up by the CB-TENG. (B) Photograph of hygrothermograph powered by the CB-TENG. (C) The charging abilities of CB-TENG for different capacitors. (D) The CB-TENG in the operating state of the instrument surface vibration drive to achieve the instrument frequency monitoring.

TENG is used as a high power supply to provide energy directly to the device. As shown in Figure 4A and Video S4, it can simultaneously light 70 LEDs at 33 Hz under 50 μm vibration. Furthermore, after 26 s of charging, it can power a hygrothermograph under the same conditions (Figure 4B and Video S5). As shown in Figure 4C, the charging abilities of CB-TENG for different capacitors (100 μF, 220 μF, 470 μF, and 1 mF) are analyzed and compared at 33 Hz, 50 μm vibration conditions. As the capacitance value increases, the charging speed slows down and the voltage also decreases. The CB-TENG can charge a 100 μF capacitor to 4 V in 77 s and a 1 mF capacitor to 0.7 V in 100 s, It shows that CB-TENG is able to drive small electronic devices. Figure 4D shows the CB-TENG in the operating state of the instrument surface vibration drive to achieve the instrument frequency monitoring. The resonant frequency of the CB-TENG is adjusted by counterweights to match the working instrument. When the instrument is in normal operation, the working light and the status light can be light at the same time through the circuit control; when the instrument failure occurs, its operating frequency changes, the CB-TENG output decreases, the circuit triode is in the cutoff state, and only the working light can be light; When the instrument is in the off state, the CB-TENG does not work and the lights are not on. CB-TENG enables frequent monitoring of the instrument’s operating status.

Video S4. CB-TENG light 70 LEDs

Video S5. CB-TENG power a hygrothermograph

## Discussion

In summary, we developed a cantilever-beam-structured TENG for efficient low-frequency micro-vibration energy harvesting. The cantilever beam structure amplifies micro-vibration energy, improving collection efficiency. A quantitative comparison with a piezoelectric bimorph shows the CB-TENG delivers higher output power in the 30–90 μm micro-vibration range. The effects of cantilever beam length and contact distance were systematically investigated. A maximum peak power of 2.685 mW was achieved at 33 Hz with beam lengths of 30–50 mm and contact distances of 2.5 and 5.0 mm, sufficient to power equipment condition monitoring and sensing networks. This work provides a constructive solution for efficient vibration energy harvesting and contributes to expanding energy sources for small-displacement applications.

### Limitations of the study

The core design of CB-TENG relies on mechanical resonance to achieve displacement amplification, resulting in its high-performance output being strictly concentrated within a narrow frequency bandwidth (around 33 Hz). Output power drops sharply once the excitation frequency deviates from the resonance point. This characteristic enables outstanding performance in vibration environments with a single, stable dominant frequency, but makes it difficult to effectively harvest energy from broadband or frequency-varying environmental vibrations. This represents a common challenge faced by resonance-based energy harvesters.

To address these limitations, future research will focus on developing nonlinear or broadband cantilever beam structures to expand the operational bandwidth. Through these efforts, the goal is to advance CB-TENG from laboratory prototypes to self-powered monitoring solutions in real industrial environments.

## Resource availability

### Lead contact

Further information and requests for resources and reagents should be directed to and will be fulfilled by the lead contact, Hengyu Guo (physghy@cqu.edu.cn).

### Materials availability

Materials used in the study are commercially available.

### Data and code availability

All data reported in this paper will be shared by the lead contact upon reasonable request. Hengyu Guo (physghy@cqu.edu.cn).

No new code was generated during the course of this study.

Any additional information required to reanalyze the data reported in this paper is available from the lead contact upon reasonable request.

## Acknowledgments

This work is financially supported by the 10.13039/501100001809NSFC (52302219 and 52572204), Science and Technology Research Program of 10.13039/501100007957Chongqing Municipal Education Commission (KJZD-K202500505), 10.13039/501100005230Natural Science Foundation of Chongqing (CSTB2025NSCQ-GPX1032).

## Author contributions

W.C. and H.G. conceived and designed the project.

W.C. and H.G. wrote the manuscript.

## Declaration of interests

The authors declare no conflicts of interest for this work.

## STAR★Methods

### Key resources table

**Table:** 

REAGENT or RESOURCE	SOURCE	IDENTIFIER
**Chemicals, peptides, and recombinant proteins**		

Spring Steel Plate	Kobetool, Germany, width 30 mm, length 80 mm, thickness	N/A
FEP film	Shanghai Witlan Industry Co., Ltd	N/A

### Experimental model and study participant details

This study does not use experimental methods typical in the life sciences.

### Method details

#### Fabrication of the CB-TENG components

CB-TENG Components was mainly composed of two parts, Which consists of a spring steel plate cantilever beam and two bracket model.

Commercial spring steel plate (Kobetool, Germany, width 30 mm, length 80 mm, thickness 0.15 mm, the surface of the steel sheet is evenly perforated, and the diameter of the hole is 1 mm).

The bracket model is printed by 3D printer, and the printing material is ABS. 0.01 mm Copper tape is closely covered on the bracket model.

Commercial FEP film (30 μm) as a dielectric layer, closely covering the Copper tape.

### Quantification and statistical analysis

The CB-TENG was driven by the actuating vibration table (SA-JZ0057). The function signal generator (UNI-T UTG932) transmits the signal to the actuating vibration table through the power amplifier (SA-PA010), which causes the exciter to vibrate according to the signal.

The output of the CB-TENG and piezoelectric bimorph were tested by an electrometer (Keithley 6514) with a Data Acquisition Card (NI PCI-6259).

The spring steel sheet was clamped at one end to form a cantilever, with the clamp rigidly mounted on the shaker table. The other (free) end was placed between two stationary FEP-coated electrodes, with the gap distance precisely set. A proof mass was attached near the free end to tune the first resonant frequency to 33 Hz. The shaker provided base excitation to the fixed end of the cantilever, simulating environmental micro-vibrations.

### Additional resources

This study has not generated or contributed to a new website/forum.

## Supplemental information

Document S1. Figures S1–S13, and Method S1
