# Supplementary material for: A cantilever-beam-structured triboelectric nanogenerator for micro-vibration energy harvesting
Source: iScience. 2026 Apr 13;29(5):115690. doi: 10.1016/j.isci.2026.115690 (PMC13138215; doi:10.1016/j.isci.2026.115690)
Supplement: Document S1. Figures S1–S13, and Method S1 [file mmc1.pdf]

iScience, Volume *29*

Supplemental information

A cantilever-beam-structured triboelectric nanogenerator for micro-vibration energy harvesting

Wenxuan Chang and Hengyu Guo

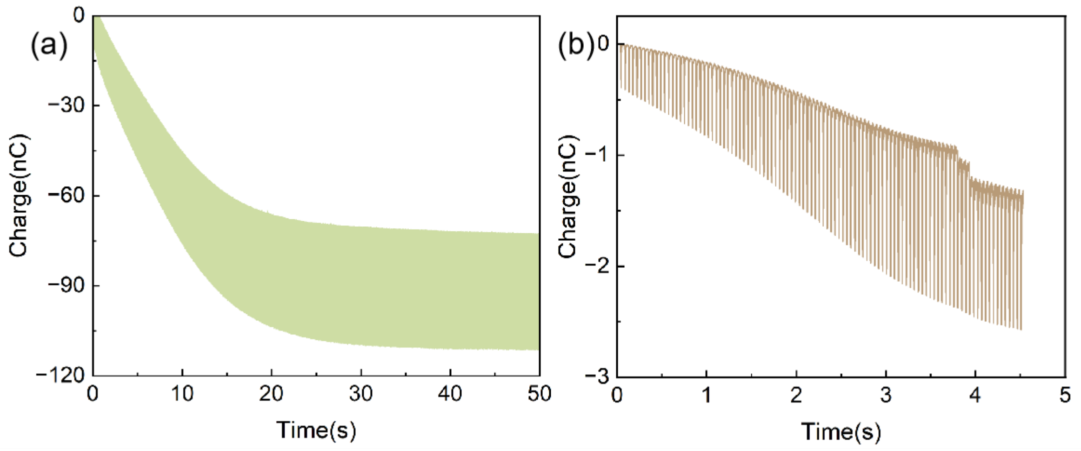

Figure S1. Variation of charge under different the spring steel sheet. (a)no perforating holes the spring steel sheet. (b)perforating holes in the spring steel sheet.

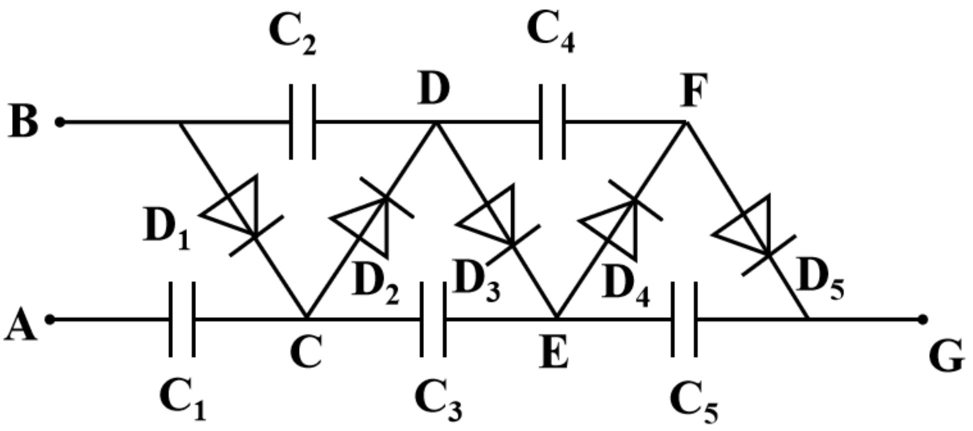

Figure S2. The voltage multiplying circuit diagram.

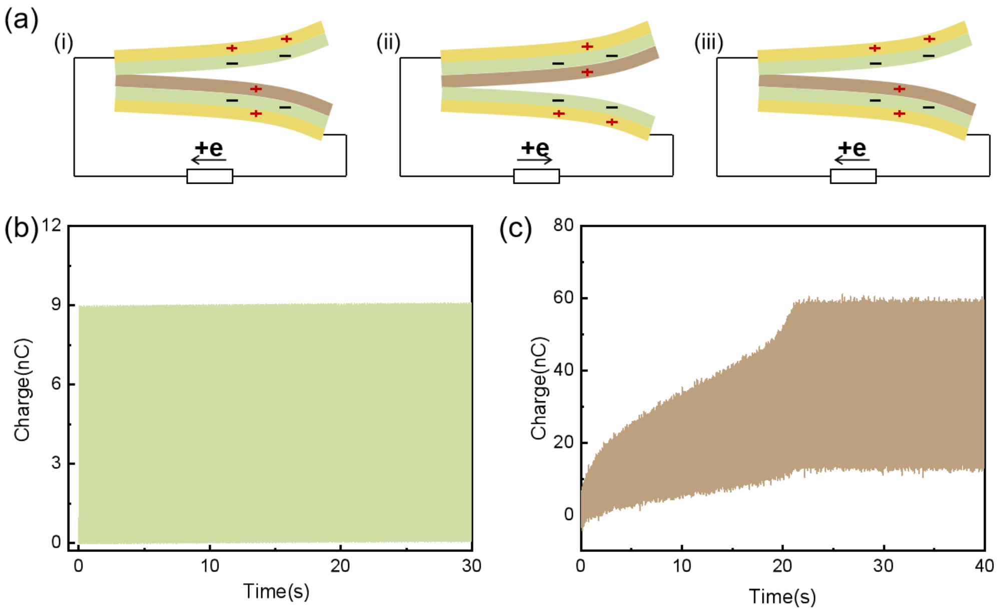

Figure S3. (a) The working mechanism of CB-TENG without VMC unit. The charge of CB-TENG at different circuit connections (b) no VMC unit and (c) with VMC unit.

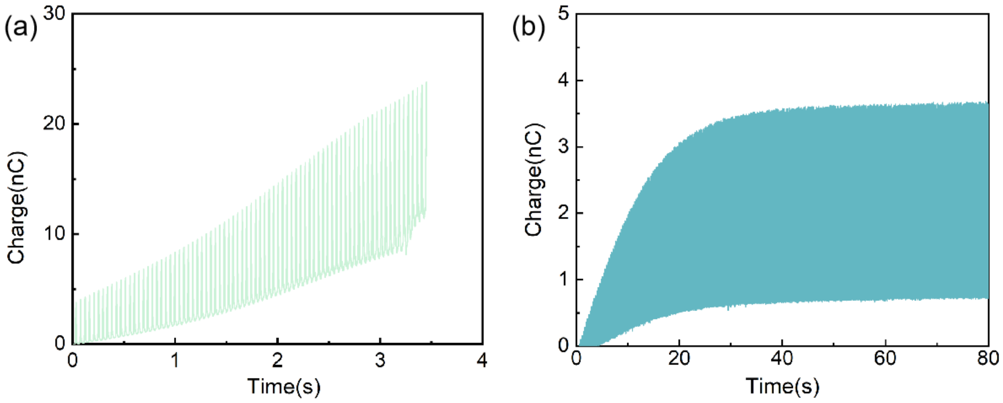

Figure S4. The charge with the vibration conditions (2.0(a) and 7.5(b) mm).

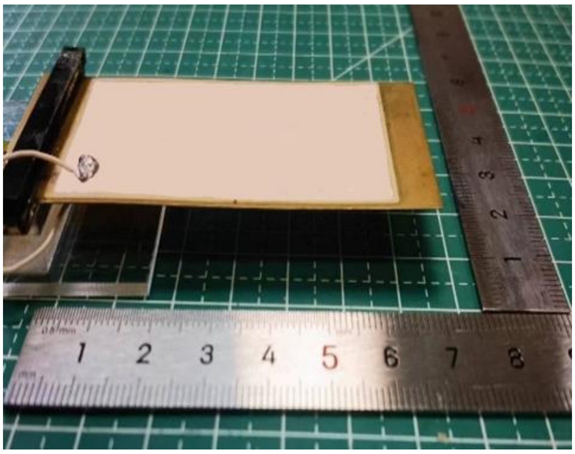

Figure S5. The photograph of a piezoelectric bimorp.

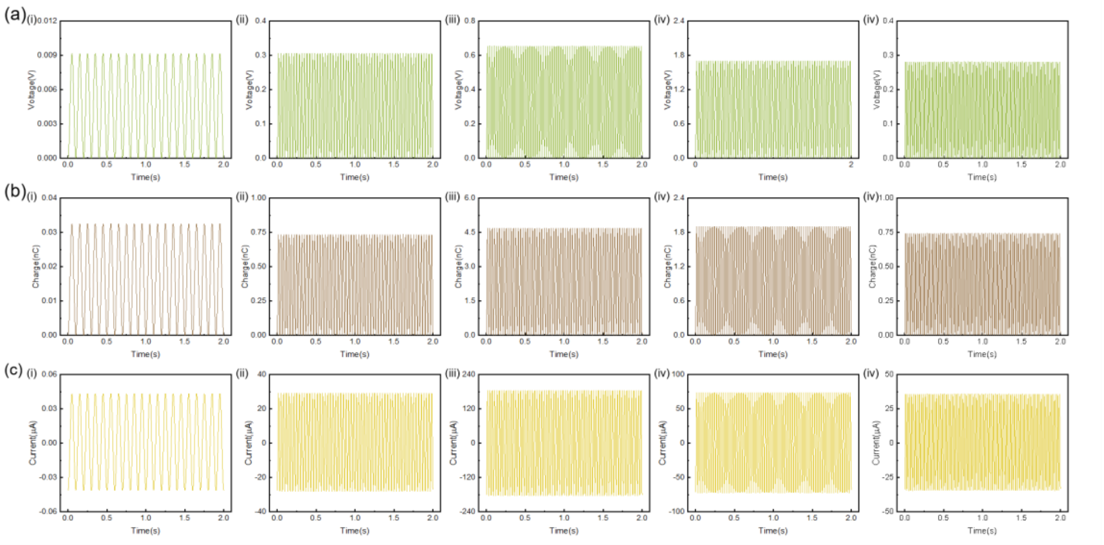

Figure S6. The performance of (a) Voltage, (b) Charge and (c) Current of piezoelectric bimorph at different frequencies (i) 10 Hz. (ii) 30 Hz. (iii) 33 Hz. (iv) 35Hz. (v) 40 Hz, respectively.

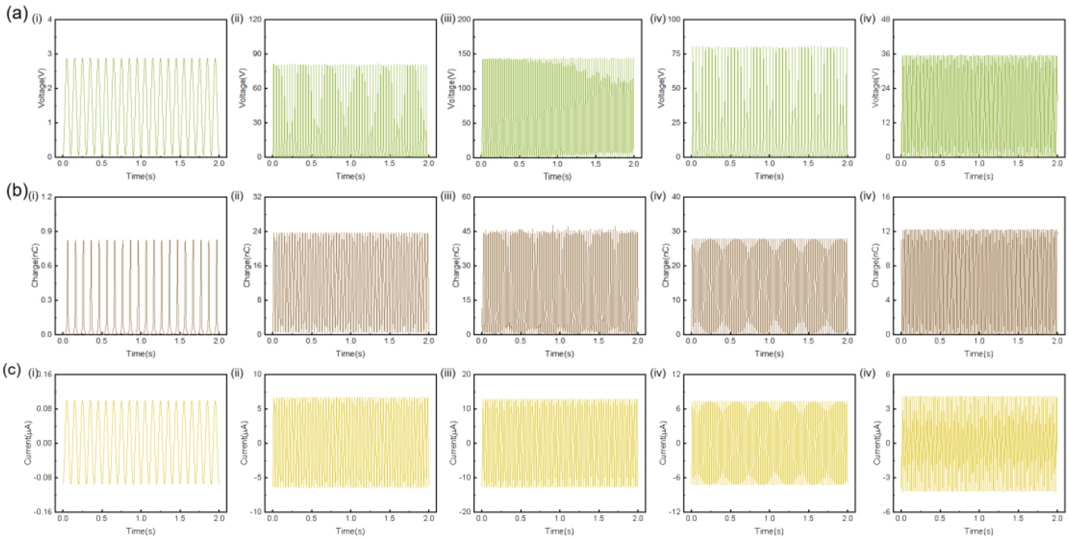

Figure S7. The performance at stable state of (a) Voltage, (b) Charge and (c) Current of CB-TENG at different frequencies (i) 10 Hz. (ii) 30 Hz. (iii) 33 Hz. (iv) 35Hz. (v) 40 Hz, respectively.

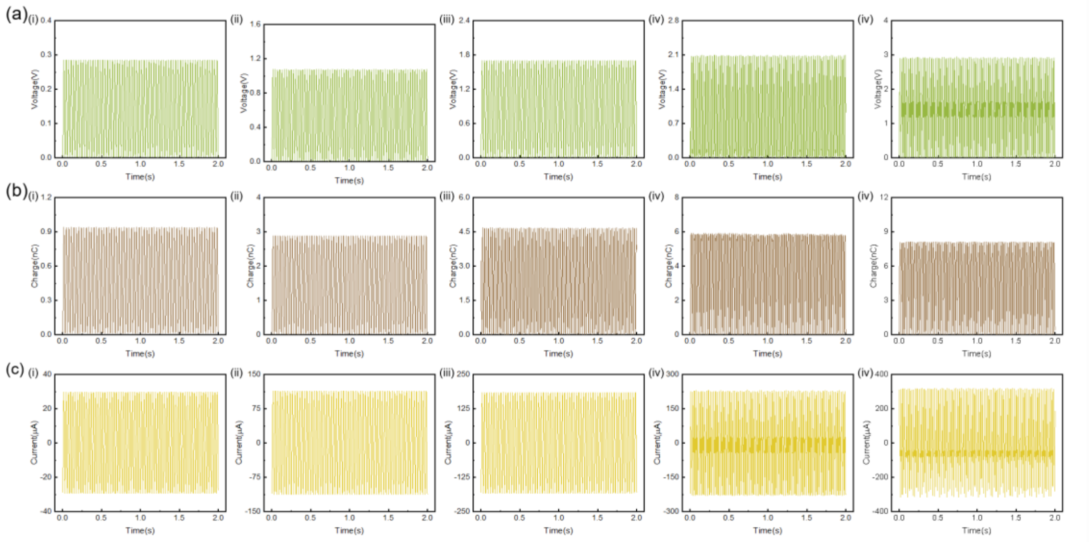

Figure S8. The performance of (a) Voltage, (b) Charge and (c) Current of piezoelectric bimorph at different vibration amplitude (i) 10 mm. (ii) 30 mm. (iii) 50 mm. (iv) 70 mm. (v) 90 mm, respectively.

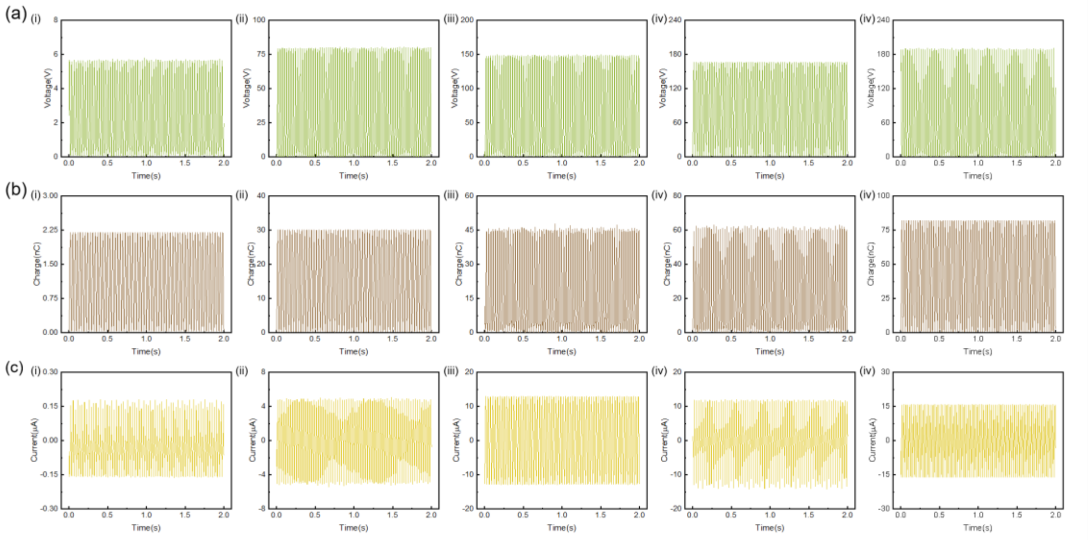

Figure S9. The performance at stable state of (a) Voltage, (b) Charge and (c) Current of CB-TENG at different vibration amplitude (i) 10 mm. (ii) 30 mm. (iii) 50 mm. (iv) 70 mm. (v) 90 mm, respectively.

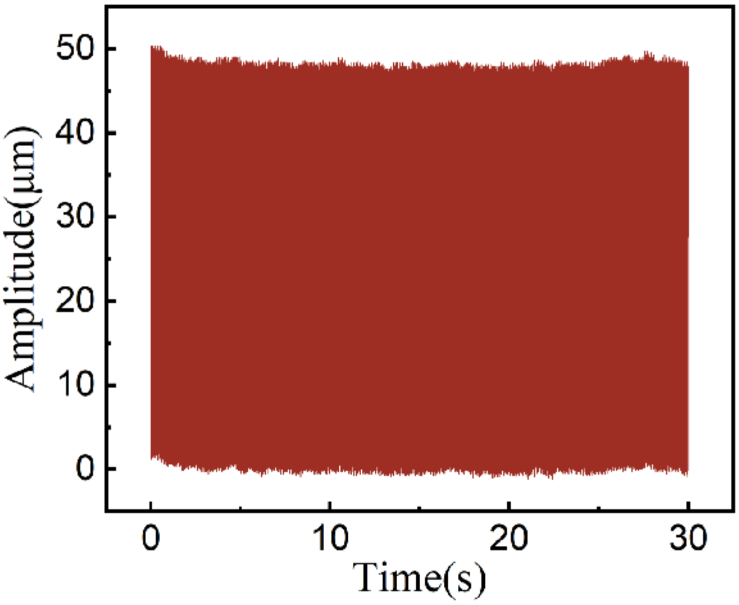

Figure S10. The vibration amplitude of the actuating vibration table is adjusted to 50 μm by laser ranging.

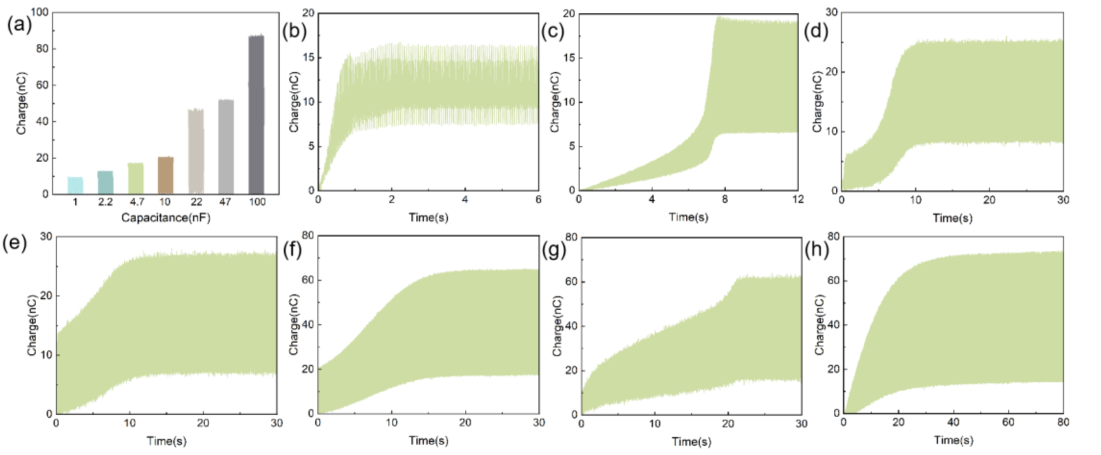

Fig S11. The Charge accumulation curves at different capacitance values (a) 1nF (b) 2.2nF (c)4.7nF (d) 10nF (e) 22nF (f) 47nF (g) and 100 nF (h).

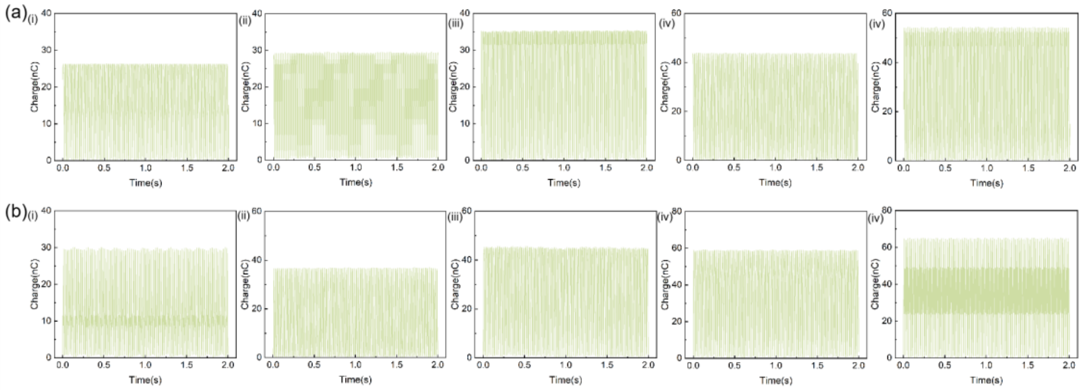

Fig S12. The performance at stable state of (a) Voltage, (b) Charge and (c) Current of CB-TENG at different frequencies (i) 10 Hz. (ii) 30 Hz. (iii) 33 Hz. (iv) 35Hz. (v) 40 Hz, respectively.

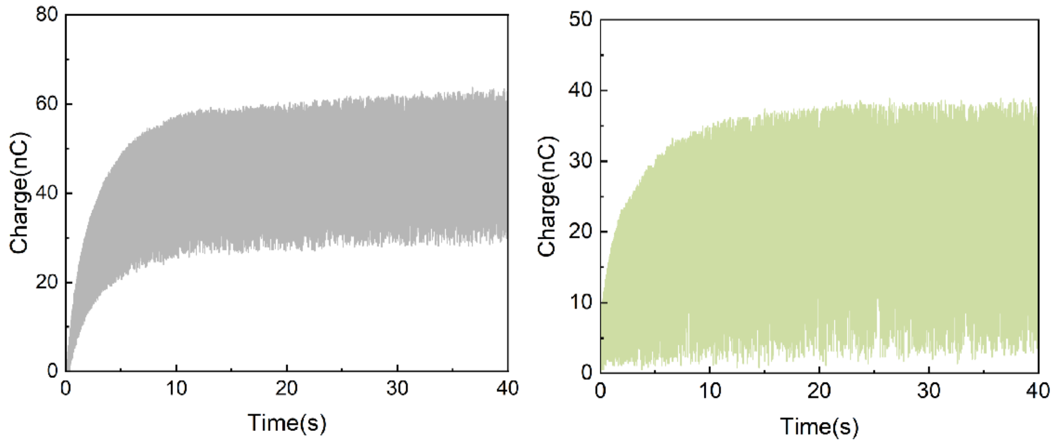

Fig S13. The charge with different materials. (a) Kapton. (b) PTFE.

Note S1

When TENG is in contact with the upper dielectric layer, U_{TENG} = -U_{AB}, diode D_1 conducts, the rest of the diodes are cut off, capacitor C_1 is charged, and the voltage across its terminals U_{C1} = U_{CA} = U_{AB} after charging is completed. When TENG is in contact with the lower dielectric layer, U_{TENG} = U_{AB}, and the voltage across capacitor C_1 is unable to change abruptly, U_{CB} = 2*U_{AB}, so diode D_2 is on, the rest of the diodes are cut off, capacitor C_2 is charged, and the voltage across its terminals U_{C2} = U_{DB} = 2*U_{AB} after charging is completed. When TENG contacts the upper dielectric layer again, U_{TENG} = -U_{AB}, while following the principle that the voltage at both ends of the capacitor unable change suddenly, U_{DA}=U_{DB}+U_{AB}=3*U_{AB}, so D_1 and D_3 are on, the rest of the diodes are cut off, and the capacitors C_1 and C_3 are charged, after the charging is completed, the voltage at both ends of C_1 is U_{C1} = U_{AB}, the voltage at both ends of C_3 is U_{C3}=2*U_{AB}, and U_{EA}=3*U_{AB}; TENG contact with the lower dielectric layer again, U_{TENG} = U_{AB}, while following the principle that the voltage at both ends of the capacitor cannot change abruptly, U_{EB} = 4*U_{AB}, so diodes D_2 and D_4 are on, the rest of the diodes are cut off, capacitors C_2 and C_4 are charged, the voltage at both ends of the capacitor U_{C2} = 2*U_{AB}, U_{C4} = U_{FD} = 2*U_{AB}, U_{FB} = 4*U_{AB} after charging is completed. When TENG continues to contact with the upper dielectric layer, U_{TENG} = -U_{AB}, while following the principle that the voltage at both ends of the capacitor cannot change abruptly, U_{FA} = U_{FD} + U_{DB} + U_{BA} = 5*U_{AB}, so D_1, D_3, D_5 are on, the rest of the diodes are cut off, the capacitors C_1, C_3, C_5 are charged, after charging is completed, the voltage at both ends of C_5 is U_{C5} =2*U_{AB}, so U_{GA}=5*U_{AB}. In the subsequent AC voltage cycle changes, the power is continuously supplied to the post-stage circuit, and finally the output voltage is stabilized.
